# Volumetric Response beyond Six Months of Cardiac Resynchronization Therapy and Clinical Outcome

**DOI:** 10.1371/journal.pone.0124323

**Published:** 2015-05-01

**Authors:** Jetske van ’t Sant, Aernoud T. L. Fiolet, Iris A. H. ter Horst, Maarten J. Cramer, Mirjam H. Mastenbroek, Wouter M. van Everdingen, Thomas P. Mast, Pieter A. Doevendans, Henneke Versteeg, Mathias Meine

**Affiliations:** 1 Department of Cardiology, University Medical Center Utrecht, Utrecht, The Netherlands; 2 Department of Medical and Clinical Psychology, CoRPS—Center of Research on Psychology in Somatic diseases, Tilburg University, Tilburg, The Netherlands; University of Minnesota, UNITED STATES

## Abstract

**Aims:**

Response to cardiac resynchronization therapy (CRT) is often assessed six months after implantation. Our objective was to assess the number of patients changing from responder to non-responder between six and 14 months, so-called late non-responders, and compare them to patients who were responder both at six and 14 months, so-called stable responders. Furthermore, we assessed predictive values of six and 14-month response concerning clinical outcome.

**Methods:**

105 patients eligible for CRT were enrolled. Clinical, laboratory, ECG, and echocardiographic parameters and patient-reported health status (Kansas City Cardiomyopathy Questionnaire [KCCQ]) were assessed before, and six and 14 months after implantation. Response was defined as ≥15% LVESV decrease as compared to baseline. Major adverse cardiac events (MACE) were registered until 24 months after implantation. Predictive values of six and 14-month response for MACE were examined.

**Results:**

In total, 75 (71%) patients were six-month responders of which 12 (16%) patients became late non-responder. At baseline, late non-responders more often had ischemic cardiomyopathy and atrial fibrillation, higher BNP and less dyssynchrony compared to stable responders. At six months, late non-responders showed significantly less LVESV decrease, and higher creatinine levels. Mean KCCQ scores of late non-responders were lower than those of stable responders at every time point, with the difference being significant at 14 months. The 14 months response was a better predictor of MACE than six months response.

**Conclusions:**

The assessment of treatment outcomes after six months of CRT could be premature and response rates beyond might better correlate to long-term clinical outcome.

## Introduction

Cardiac resynchronization therapy (CRT) is an established treatment for patients with congestive heart failure (CHF) and a wide QRS complex.[1] A common measure for determining a patient’s response to CRT is the decrease in left ventricular end systolic volume (LVESV) six months after device implantation.[2] Patients demonstrating ≥15% LVESV decrease are classified as responder; otherwise they are classified as non-responder.[2,3] In multi-center studies it has previously been demonstrated that this reverse remodeling is a process which continues until 18–24 months after device implantation.[4,5] Due to continuous reverse remodeling, initial non-responders (<15% LVESV decrease) may become responders at a later time (late responders), while initial responders (≥15% LVESV) may later become non-responders due to, possibly, diminishing beneficial effects of CRT over time.

At present, many studies and clinicians evaluate CRT response within six months after device implantation and focus on pre-implantation factors predicting this response. However, limited data are available concerning the prevalence and predictors of long-term changes in response to CRT. Therefore, in the current study we assessed the number and characteristics of patients whose response at 14 months differed from their response at six months. Our main focus was on late non-responders as we hypothesize that these might have a worse prognosis than (late and stable) responders and should therefore be identified. Hence, we also examined the correlation of 14 months response with health outcomes, including patient-reported health status and major adverse cardiac events (MACE).

## Methods

### Study design and cohort

This was a prospective, single center study designed to study the influence of PSYchological factors on health outcomes in HEART failure patients treated with CRT (PSYHEART-CRT). Patients eligible to CRT, according to applicable guidelines and evidence-based medicine at time of inclusion, were enrolled between January 2009 and August 2011 at the University Medical Center Utrecht (UMCU).

### Ethics statement

The study was conducted in accordance with the Declaration of Helsinki, and the study protocol was approved by the local Medical Ethics Committee of the UMCU (protocol number 08–246) and patients signed informed consent. A more extensive description has been published previously.[6]

### Echocardiography

Echocardiographic studies were performed prior to implantation (baseline), and six and 14 months after device implantation. Data were acquired using Philips IE 33 (Philips Medical Systems, Andover, Massachusetts, USA) or Vivid 7 (General Electric, Milwaukee, USA) ultrasound machines. Apart from speckle tracking analysis, echocardiographic parameters were assessed offline using Xcelera software (R3.3L1). Speckle tracking was performed for studies on the Vivid 7 and analyzed using EchoPac software (version 11.2, revision 1.1). Volumes and other measurements were assessed by one observer and in accordance with the guidelines of the American Society of Echocardiography (ASE) and European Association of Echocardiography (EAE).[7] Measurements were performed on three separate beats, or five beats in case of irregular rhythms.

Mitral regurgitation at baseline and after six months was visually assessed and extracted from echocardiographic records.

### Volume response

LVESV was assessed by Simpsons’ biplane method. Volume changes were assessed between baseline and six months FU, baseline and 14 months FU and between six and 14 months FU.

Response to CRT was defined as relative decrease in LVESV of ≥15%, which has been shown to predict clinical outcome up to five years after CRT implantation.[8] Non-responders were patients demonstrating <15% LVESV decrease, or who died due to heart failure or received a left ventricular assist device (LVAD). Response rates were assessed at six and 14 months after CRT implantation. Patients who were responder at both six and 14-month follow-up (FU) were termed ‘stable responders’. Six-month responders turning into non-responders at 14 months were termed ‘late non-responders’. Six-month non-responders, turning into responders at 14 months were termed ‘late responders’. Six and 14-month non-responders were termed ‘stable non-responders’.

### Dyssynchrony measurements

Doppler flows over the pulmonary and aortic valve were recorded and time from Q to onset of flow was assessed for both valves.[2] Interventricular mechanical delay (IVMD) was defined as the time span between the opening of the aortic valve and the pulmonary valve.

ΔIVMD was assessed between baseline and six months FU.

Systolic rebound stretch of the septum (SRS_sept_) was evaluated using speckle tracking by evaluation of longitudinal septal strain, as previously described.[3,9] Frame rates were kept between 50–110 frames per second. Systole was defined as the period from mitral valve closure up to aortic valve closure as assessed by pulsed Doppler waves over the mitral and aortic valve, respectively. ΔSRS_sept_ was assessed between baseline and six months FU.

### Demographic, clinical, ECG, and laboratory variables

Demographic, clinical, ECG, and laboratory variables were extracted from patients’ medical records, as described previously.[6] Definition of left bundle branch block (LBBB) was conform current American Heart Association/American College of Cardiology Foundation/Heart Rhythm Society (AHA/ACCF/HRS) recommendations.[10] Pacing percentages were derived through device interrogation.

### Patient-reported health status

At baseline and at six and 14-month FU, patients completed the Kansas City Cardiomyopathy Questionnaire (KCCQ) to assess CHF-specific health status.[11] The KCCQ is a 23-item, self-report questionnaire that quantifies physical limitation, symptoms, social function and quality of life of patients with CHF. These four health status subscales can be combined into a single overall summary score. Scores are transformed into a score ranging from 0 to 100 with higher scores representing better health status. The validity and reliability of the KCCQ have previously been established and this method has been shown to be highly sensitive to clinical change in CHF patients.[11]

### Major adverse cardiac events

MACE cases were defined as hospitalization due to heart failure, LVAD implantation, heart transplantation or death due to heart failure. Assessment took place for up to 24 months after CRT implantation.

### Statistical analyses

Statistical analysis was performed using SPSS version 20.0 (SPSS Inc., Chicago, Illinois). Continuous variables are presented as mean with standard deviation (SD) when normally distributed and as median with interquartile range (IQR) in case of non-normal distribution. Categorical variables are presented as numbers and percentages. Differences at baseline and at six months FU between stable responders and late non-responders were assessed. Categorical variables were compared using Pearson’s Chi-square and continuous variables were assessed using students T-tests or Mann-Whitney U, as appropriate. Related samples of continuous variables were assessed using students T-tests or Friedman’s two-way analysis of variance by ranks test. Related samples of categorical variables were assessed with McNemar. Furthermore, the correlation between response rates, at six and 14 months, and MACE was assessed and compared with Pearson’s Chi-square and net reclassification index (NRI).[12] The NRI is a measure demonstrating the improvement in risk prediction from (in this case) 14 months response rates over six months response rates. Calculation of NRI was based on the following categories of chances of becoming a responder: <0.33, 0.33–0.66, and >0.66. To measure the correlation between response rates and MACE, solely patients with six and 14-month echocardiographic studies were taken into account. Non-responders by other definition than <15% LVESV decrease; either receiving an LVAD or death due to heart failure, were excluded for this analysis.

A sensitivity analysis was performed for a subsample thereby excluding patients with atrial fibrillation (AF) as this is associated with reduced CRT response. [13–15]

## Results

Of 139 patients that consented to participate in the study, 12 lacked a baseline echocardiographic study, 11 cases had insufficient image quality, nine were lost to follow-up, two died of non-cardiac cause.

**Fig 1:**
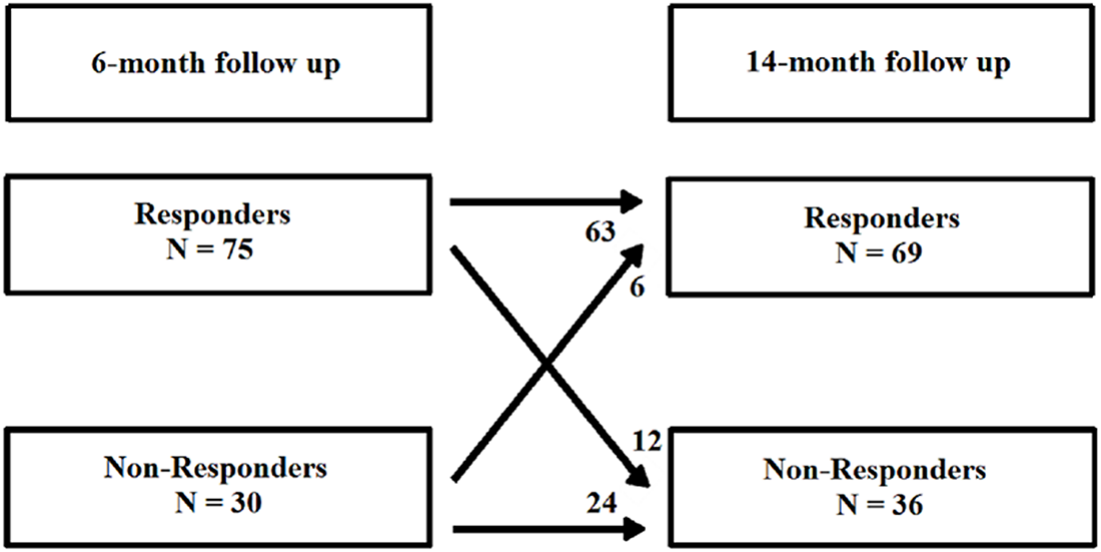
Flow chart of responders and non-responders.

**Fig 2:**
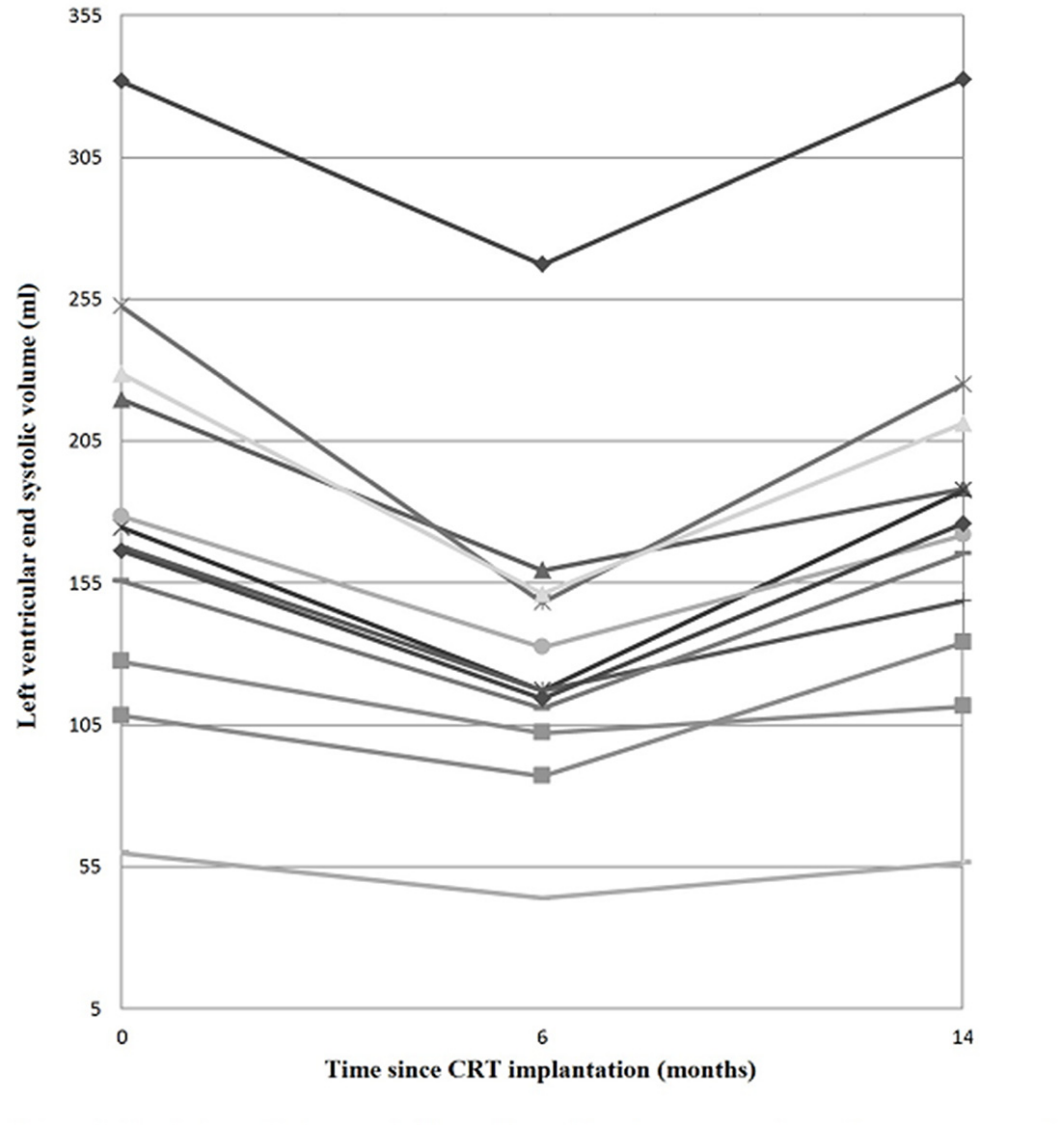
Evolution of Left Ventricular End Systolic Volume over time of late non-responders. At six-month follow-up late non-responders showed a significant median decrease of left ventricular end systolic volume (LVESV) of 28%. However, at 14-month follow-up, LVESV almost returned to baseline values again.

Six months after CRT implantation 71% (n = 75) patients were responders and 29% (n = 30) were non-responders. Of these responders 84% (n = 63) were stable responders and 16% (n = 12) became late non-responders, as shown in Fig 1. Fig 2 demonstrates the evolution of LVESV for late non-responders. Of the six-month non-responders, 80% (n = 24) were stable non-responders and 20% (n = 6) became late responders.

At baseline, six and 14 months FU, 15 patients demonstrated AF at least during one assessment. Of these, three had permanent AF, six had persistent AF (for which one a His-ablation was perfomed), and three had paroxysmal AF. Three patients had AF solely at baseline, and device interrogation did not show AF anymore during follow-up. If all patients with AF were neglected, crossover from response to late non-response still occurred in 12% of the six-month responders.

### Six-month responders

Baseline characteristics of the 75 six-month responders, stratified by stable responders and late non-responders, are shown in Table 1. New York Heart Association (NYHA) functional classification did not differ significantly between stable responders and late non-responders and the majority (77%) was in NYHA class II. LBBB was present in 59% of the patients, interventricular conduction delay (IVCD) in 27%. Fourteen percent of patients were paced in the right ventricle (RV). Mean left ventricular ejection fraction (LVEF) was 25±9%. These numbers did not differ significantly between stable responders and late non-responders.

**Table 1:** Baseline data of six-month responders, and stratified by late non-responders and stable responders.

	All six-month responders (n = 75)	Late non-responders (n = 12)	Stable responders (n = 63)
**Baseline clinical data**			
Age, years, mean±SD	65.4±10.5	70.6±7.0	64.6±11.0
Male (%)	50 (67)	11 (92) *	39 (62) *
NYHA II (%)	15 (20)	1 (8.5)	14 (22)
NYHA III (%)	58 (77)	10 (83)	48 (76)
NYHA IV (%)	2 (3)	1 (8.5)	1 (2)
Ischemic cardiomyopathy, (%)	33 (44)	10 (83) *	23 (37) *
**Baseline ECG data**			
QRS duration, ms, mean±SD	165±25	160±26	166±25
LBBB (%)	44(59)	6(50)	38(60)
IVCD (%)	20(27)	4(33)	16(25)
RBBB (%)	1(1)	0(0)	1(2)
RV pacing (%)	10(13)	2(17)	8(13)
Atrial fibrillation (%)	11(15)	4(33) *	7(11) *
**Baseline Medication**			
ACE inhibitor/ AT2-antagonist (%)	65(89)	10(83)	55(89)
Diuretics (%)	59(81)	10(83)	49(80)
Beta-blocker (%)	58(77)	9(75)	49(80)
**Baseline laboratory data**			
Creatinine, μmol/L, median (IQR)	112(33)	119(33)	106(45)
BNP, pmol/L, median (IQR)	52(84)	113(341) *	65(101) *
**Baseline echocardiographic data**			
LVEF, %, mean±SD	25±9	24±10	25±8
LVESV, ml, median (IQR)	166(65)	171(92)	160(64)
IVMD, ms, mean±SD	44±28	21±14^#^	48±28^#^
SRSsept, %, median (IQR)	4.31(3.89)	0.57(2.8) ^#^	4.52(3.7) ^#^
Tapse, cm, mean±SD	1.8±0.5	1.5±0.3*	1.9±0.5*
RV peak systolic velocity, cm/sec, median (IQR)	10.0(4.15)	8.5(1.9)	10.3(4.1)
LA volume, ml/m^2^, median (IQR)	43.4(20.2)	49.7(20.9)	43.2(20.8)
RA area, cm^2^, median (IQR)	15(8)	19(7) *	15(7) *
E/E’, median (IQR)	13(9)	15(11)	13(8)
Moderate or severe mitral regurgitation, n (%)	5 (7)	1 (8)	4 (6)

p-value between late non-responders and stable responders:

* = p < 0.05,

^#^ = p < 0.001

ACE: angiotensin-converting enzyme, BNP: B-type natriuretic peptide, IQR: Interquartile range, IVCD: Interventricular conduction delay, IVMD: Interventricular mechanical delay, LA: Left atrium, LBBB: Left bundle branch block, LVEDV: left ventricular end diastolic volume, LVEF: left ventricular ejection fraction, LVESV: left ventricular end systolic volume, NYHA: New York Heart Association, RA: Right atrium, RBBB: Right bundle branch block, RV: Right ventricle, SRS_sept_: Systolic Rebound Stretch of the Septum

### Stable responders versus late non-responders

#### Baseline

As shown in Table 1, late non-responders were more often male (92% vs 62%) and more likely to have ischemic cardiomyopathy (ICM; 83% vs 37%) as compared to stable responders. Elapsed time between last myocardial infarction and last invasive treatment (percutaneous coronary intervention (PCI) or coronary artery bypass surgery) for coronary artery disease did not differ significantly between stable responders and late non-responders with ICM (7±7 years; results not shown). Late non-responders more often showed AF compared to stable responders. Furthermore, late non-responders showed significantly lower IVMD and SRS_sept_, and higher B-type natriuretic peptide (BNP) levels compared to stable responders.

If patients with AF at baseline were excluded from the analysis, baseline differences between late non-responders and stable responders did not change, except for RV peak systolic velocity which appeared to be significantly lower in non-responders.

#### Six-month follow-up

An overview of the six-month FU data of stable responders and late non-responders is provided in Table 2. Late non-responders showed significantly higher LVESV at six months than stable responders (117 ml vs 88 ml), and significantly lower LVEF (29% vs 35%). Absolute LVESV decrease in the first six months of CRT did not differ significantly between both groups. However, relative LVESV decrease was significantly lower for late non-responders than for stable responders. Creatinine and BNP levels were higher in late non-responders than stable responders. In addition, late non-responders had a lower biventricular pacing percentage and occurrence of AF was significantly higher as compared to stable responders after six months.

**Table 2:** Six-month follow-up data of late non-responders and stable responders.

	Late non-responders (n = 12)	Stable responders (n = 63)
**Six-month FU clinical data**		
NYHA I (%)	0 (0)	7 (11)
NYHA II (%)	7(58)	40(64)
NYHA III (%)	5(42)	16(25)
Pacing percentage, %, median (IQR)	96 (7) *	99 (5) *
**Six-month FU ECG data**		
Stimulated QRS duration, ms, mean±SD	147±23	144±20
Left to right axis shift (%)	6(50)	32(53)
Atrial fibrillation (%)	3(25) *	4(8) *
**Six-month FU medication**		
ACE inhibitor/AT2 antagonist (%)	10(83)	52(88)
Diuretics (%)	10(83)	44(74)
Beta-blocker (%)	9(75)	50(85)
Statines (%)	10(83) *	28(48) *
**Six-month FU laboratory data**		
Creatinine, μmol/L, median (IQR)	133(88) *	107(40) *
ΔCreatinine, μmol/L, median (IQR)	13(33)	4(22)
BNP, pmol/L, median (IQR)	152(237) *	42(66) *
ΔBNP, pmol/L, median (IQR)	15(303)	-13(65)
**Six-month FU echocardiographic data**		
LVEF, %, mean±SD	29±7 *	35±9 *
Absolute ΔLVEF, %, mean±SD	5.0±9.4 *	10.7±8.0 *
LVESV, ml, median (IQR)	117(46) *	88(61) *
Relative ΔLVESV, %, median (IQR)	-28(11) *	-39(25) *
IVMD, ms, mean±SD	3±32 *	20±23 *
Absolute Δ IVMD, ms, mean±SD	-18±34	-26±29
SRSsept, %, median (IQR)	0.03(0.16) *	0.31(1.38) *
Absolute ΔSRSsept, %, median (IQR)	-0.38(2.78) *	-3.41(4.69) *
Tapse, cm, mean±SD	1.6±0.4	1.8±0.5
RV peak systolic velocity, cm/sec, median (IQR)	9.1±1.4	9.5±4.3
LA volume, ml/m^2^, median (IQR)	52(32) *	36(18) *
RA area, cm^2^, median (IQR)	16(9)	15(6)
E/E’, median (IQR)	14(6)	12(11)
Moderate or severe mitral regurgitation, n (%)	0(0)	1(2)

P-value between late non-responders and stable responders:

* = p < 0.05,

^#^ = p < 0.001

ACE: angiotensin-converting enzyme, BNP: B-type natriuretic peptide, IVCD: Interventricular conduction delay, IVMD: Interventricular mechanical delay, LA: Left atrium, LBBB: Left bundle branch block, LVEDV: left ventricular end diastolic volume, LVEF: left ventricular ejection fraction, LVESV: left ventricular end systolic volume, NYHA: New York Heart Association, RA: Right atrium, RBBB: Right bundle branch block, RV: Right ventricle, SRS_sept_: Systolic Rebound Stretch of the Septum

If patients with AF at six months were disregarded in the analyses, six-month results showed only minor changes. Differences between late non-responders and stable responders did not differ, except for pacing percentage, ΔLVEF and SRS_sept_ which did not show a significant difference anymore between late non-responders and stable responders.

#### Patient-reported health status

In total, 91% (68/75) of the six-month responders completed the KCCQ three times; at baseline, and at six and 14-month FU. At each assessment, the nine late non-responders reported a lower mean health status score than the 59 stable responders (i.e., 48.3±26.7 versus 58.5±22.3, p = 0.22 at baseline; 60.5±23.5 versus 75.9±21.7, p = 0.05 at six-month FU; and 52.9±29.0 versus 75.8±21.2, p = 0.006 at 14-month FU). This difference was statistically significant at 14 months FU only. In addition, the stable responders reported significantly increased KCCQ scores from baseline to six months FU (p<0.001), while this increase did not occur for the late non-responder group.

#### Major adverse cardiac events

Of the total population, 24% (26/105) suffered a MACE within two years after CRT implantation. For six-month responders, late non-responders and stable responders the prevalence of patients suffering a MACE were: 19% (14/75), 58% (7/12) and 11% (7/63), respectively. Table 3 demonstrates the distribution within the groups of six and 14-month responders and non-responders. The NRI increased significantly for the response rates at 14 months compared to response rates at six months: 38.1%, p = 0.009.

**Table 3:** MACE between 14–24 months after implantation in 6 and 14-month responders and non-responders.

	Six-month responders (n = 75)	Six-month non-responders (n = 23)	P-value
**MACE (%)**	14 (19)	5 (22)	0.774
**No MACE (%)**	61 (81)	18 (78)	
	**14-month responders (n = 69)**	**14-month non-responders (n = 29)**	**P-value**
**MACE (%)**	7 (10)	12 (41)	<0.001
**No MACE (%)**	62 (90)	17 (59)	

MACE: Major adverse cardiac events

## Discussion

The main finding of this study was that 16% of the six-month responders turned into non-responders after 14 months of CRT. Furthermore, we found that 14-months response rates correlated significantly better with patient-reported health status and occurrence of MACE compared to six months response rates, indicating that the change from responder to non-responder has important consequences for prognosis.

### Baseline characteristics of late non-responders

Although late non-responders and stable responders were both eligible to CRT according to the guidelines, significant differences between these groups were already present prior to implantation. Pre-implantation BNP was lower in stable responders, whereas volumes did not differ significantly between late non-responders and stable responders. Lower BNP levels are associated with more reverse remodeling and better prognosis, as high BNP indicates high wall stress associated with dilated myocardium.[16,17] In addition, most late non-responders had ICM, which has been associated with less reverse remodeling.[18] This could be attributed to the presence of denser scar tissue in patients with ICM, which is unable to undergo reverse remodeling. However, progression of cardiovascular disease could also contribute to the (late) non-response. Cutlip et al.[19] demonstrated in 1228 patients who underwent PCI that the cumulative event rate (re-stenosis and new stenosis) five years after the intervention was 45% with an annual hazard rate of 8%, indicative of the progressive character of the disease. Since in our ICM patients, mean time since last coronary intervention was more than five years, it could be hypothesized that their coronary artery disease has progressed significantly. Furthermore, late non-responders showed significantly less mechanical dyssynchrony at baseline than stable responders, which has previously been associated with non-response and worse survival rates.[2,3] In the current study, besides IVMD, SRS_sept_ was used to define mechanical dyssynchrony. Our center previously demonstrated that this parameter is a good predictor of volumetric response to CRT as well as clinical outcome.[3,20] In addition, Chan et al.[9] recently demonstrated in their cohort of CRT patients that SRS_sept_ had important additional value for the identification of CRT responders. CRT aims for the correction of dyssynchrony, thereby improving ventricular functioning and reducing heart failure symptoms. Consequently, patients with underlying dyssynchrony are more likely to respond to CRT.[3,21] However, current guidelines do not support mechanical dyssynchrony measurements concerning indication setting for CRT and eligibility for CRT is based on LVEF and measurements of *electrical* dyssynchrony. Nevertheless, our study did not show significant differences concerning QRS duration or the presence of LBBB between stable responders and late non-responders; implicating that according to current guidelines they were equally suitable to receive a CRT device and a priori would have similar chances of becoming a responder. Finally, a relatively high share of late non-responders suffered from AF as compared with stable responders. AF is associated with reduced CRT response; however the mechanism remains unclear as AF could be the result of more advanced heart failure, whereas, at the same time, it can reduce biventricular capture.[13] Both advanced heart failure and decreased biventricular capture have been associated with non-response.[14,15]

### Six-month characteristics of late non-responders

Late non-responders demonstrated significantly lower volume reductions and BNP did not reduce during the first six months of CRT. This indicates that they had less benefit from CRT compared with stable responders. It has been demonstrated that less reverse remodeling is correlated with an increase in MACE.[22] Moreover, late non-responders showed significantly higher creatinine levels at six months FU. Cardiac and renal functions influence each other and even mild renal insufficiency diminishes prognosis.[23,24] This decline in prognosis arises from many unfavorable changes occurring in patients suffering from renal failure including the activated Renin Angiotensin System, inducing cardiac remodeling.[25] Fung et al. demonstrated that decline in renal function after CRT implantation correlated with higher mortality rates.[26] They stated that the decline in renal function is probably due to the natural course of this disease and that patients with renal failure might require more intensive monitoring and more aggressive treatment. Moreover, in case of renal failure, patients may not tolerate maximum doses of essential medication, which might contribute to reduced reverse remodeling.[27]

Late non-responders showed larger average left atrial volumes, more often demonstrated AF and pacing percentages were significantly lower at six months, the three of which could very well be correlated. In general, patients with AF showed a significantly lower median pacing percentage; 90% vs 99%. At 14 months, late non-responders still more often demonstrated AF than stable responders (33% vs 10%, p = 0.028), whereas median pacing percentages no longer differed significantly; 98% vs 99%, (p = 0.34). Consequently, difference in pacing percentages at six months probably may not contribute to the response conversion in late non-responders, as this improved thereafter. This might implicate a merely modest role for AF concerning the occurrence of late non-response. Especially considering the fact that crossover from response to non-response still occurred in 12% when AF patients were not taken into account. However, the difference in AF burden complicates the interpretation of the influence of AF on late non-response. In addition, the results of this subanalysis have to be interpreted with even more caution as without AF patients sample size is compressed even further.

Moreover, despite lower volume reductions, a lack of BNP reduction, higher creatinine levels, higher frequency of AF, and lower pacing percentages, the late non-responders did show relevant reverse remodeling at six months.

### Response and health outcomes

At baseline, and six and 14 months FU, late non-responders reported lower health status than stable responders, but the difference was significant at 14 months only. This finding suggests that the correlation between volume response and patient-reported health status increases after six months of CRT.[6] At 14 months, late non-responders on average scored 23 points lower on the KCCQ than stable responders, which is a difference of major importance for patients’ daily lives and their prognosis.[28] In addition, response rates at 14 months significantly improved the prediction of MACE for, at least, two years after CRT compared to six months response rates. These results indicate that response assessment after six months of CRT might be a premature moment to assess the long-term treatment effect, especially considering the fact late non-responders had a worse prognosis than stable responders.

### Clinical implications

In daily practice the effect of CRT is usually assessed six months after device implantation. However, the high number of late non-responders found in our study indicates that the long term effect of CRT is not yet visible after six months, leading to premature and possibly incorrect conclusions about patients’ response to treatment. Patients should be monitored closely after six months of CRT. Our recommendation would be to repeat ECG, echocardiography, laboratory measurements, and health status reports beyond the first year after CRT implantation, in order to be able to consider other interventions in case of deterioration, thereby improving prognosis and preventing early MACE.

### Limitations

This is a single-center study in a real-world setting with its inherent limitations. Twelve patients lacked a baseline echocardiographic study and 11 patients had insufficient image quality. In addition, the study is underpowered for multivariable analysis; however, the main focus of this paper was to assess the prevalence of late non-responders. Nevertheless, we would encourage investigating these findings in a larger cohort, in order to confirm our results and to investigate the independent determinants of late (non)response. Moreover, the amount of biventricular pacing was estimated based on the pacing percentage provided by the device. Furthermore, during AF, biventricular pacing could be overestimated because of pseudo-fusion between intrinsic conduction and pacing. Finally, our follow-up period was 14 months, as this was the time point patients came into the clinic for their regular check-up. Yet, as previously addressed, it has been shown that reverse remodeling can continue even thereafter; until 24 months after CRT implantation.[4,5] Therefore, it would be interesting to investigate how response rates develop beyond 14 months of CRT. On the other hand, longer follow-up periods have inherently higher mortality rates causing patients to be lost for analysis.

## Conclusion

We demonstrated that 16% of six-month volume responders changes into non-responders after more than 1 year of CRT. Furthermore, 14-month response had a stronger correlation with health outcomes (i.e., patient-reported health status and MACE) than six-month response, indicating that the crossover from responder to non-responder represents a relevant change in patients’ health. This knowledge is essential for daily clinical practice as well as for future research projects on CRT as it indicates that the assessment of treatment outcomes after six months of CRT might be premature.
